# Predictive risk score model for severe fever with thrombocytopenia syndrome mortality based on qSOFA and SIRS scoring system

**DOI:** 10.1186/s12879-020-05299-7

**Published:** 2020-08-12

**Authors:** Li Wang, Zhiqiang Zou, Kun Ding, Chunguo Hou

**Affiliations:** Infectious disease department, Qishan (Infectious Disease) Hospital of Yantai, 62 Huanshan Road, Zhifu District, Yantai, Shandong 264001 People’s Republic of China

**Keywords:** Severe fever with thrombocytopenia syndrome, Risk score model, qSOFA, SIRS

## Abstract

**Background:**

Severe fever with thrombocytopenia syndrome (SFTS) is a severe systemic virus infectious disease usually having multi-organ dysfunction which resembles sepsis.

**Methods:**

Data of 321 patients with laboratory-confirmed SFTS from May 2013 to July 2017 were retrospectively analyzed. Demographic and clinical characteristics, calculated quick sequential organ failure assessment (qSOFA) score and systemic inflammatory response syndrome (SIRS) criteria for survivors and nonsurvivors were compared. Independent risk factors associated with in-hospital mortality were obtained using multivariable logistic regression analysis. Risk score models containing different risk factors for mortality in stratified patients were established whose predictive values were evaluated using the area under ROC curve (AUC).

**Results:**

Of 321 patients, 87 died (27.1%). Age (*p* < 0.001) and percentage numbers of patients with qSOFA≥2 and SIRS≥2 (*p* < 0.0001) were profoundly greater in nonsurvivors than in survivors. Age, qSOFA score, SIRS score and aspartate aminotransferase (AST) were independent risk factors for mortality for all patients. qSOFA score was the only common risk factor in all patients, those age ≥ 60 years and those enrolled in the intensive care unit (ICU). A risk score model containing all these risk factors (Model1) has high predictive value for in-hospital mortality in these three groups with AUCs (95% CI): 0.919 (0.883–0.946), 0.929 (0.862–0.944) and 0.815 (0.710–0.894), respectively. A model only including age and qSOFA also has high predictive value for mortality in these groups with AUCs (95% CI): 0.872 (0.830–0.906), 0.885(0.801–0.900) and 0.865 (0.767–0.932), respectively.

**Conclusions:**

Risk models containing qSOFA have high predictive validity for SFTS mortality.

## Background

Severe fever with thrombocytopenia syndrome (SFTS) is an emerging hemorrhagic disease with high mortality of 12–30%, which is caused by the SFTS virus (SFTSV) infection [1]. The clinical manifestations typically became worse within a week of admission, and most of them had multi-organ dysfunction. Previous studies have shown many laboratory variables and clinical parameters to be associated with death including elevated levels of aspartate aminotransferase (AST), serum creatinine (sCr), blood urea nitrogen (BUN), viral loads, age and neurological symptoms [2, 3]. Study has demonstrated that the inflammatory cytokine storm was associated with the severity of SFTS [4] and specific treatment for SFTS was not proved [5]. Limited data showed that ribavirin therapy was effective only in patients with a viral load below 1 × 106 copies/mL [2]. Therefore, the initial prediction of an adverse outcome is of utmost importance for taking effective preventative and combined internal medical therapeutic measures to prevent the disease from becoming worse which can be attained by monitoring clinical and laboratory variables.

Sepsis is a life-threatening organ dysfunction caused by the dysregulated host response to infection. Sequential organ failure assessment (SOFA), quick SOFA (qSOFA) scoring systems, and systemic inflammatory response syndrome (SIRS) criteria are usually used to evaluate the severity of sepsis [6]. Though the term sepsis may not be appropriate for life-threatening acute organ dysfunction caused by nonbacterial infections, such as SFTS, some authors have suggested using the term sepsis or analogous severe infectious course in this case [7]. SOFA, qSOFA scoring systems and SIRS criteria have widely applied for the prediction of in-hospital mortality who are likely to have sepsis (or analogous severe infectious course) in the intensive care unit (ICU) [6, 8]. However, their roles have not been well-evaluated for the prediction of in-hospital mortality of SFTS patients. Though several risk models have been established for the prediction of SFTS mortality, the indices included can not be available quickly after admission rendering delay of their utility, such as viral loads [3, 9]. Compared to SOFA score of which many components were quickly unavailable in most patients, qSOFA score and SIRS criteria were composed of simple and easily obtained parameters [6]. qSOFA was defined as a score composed of three binary variables (tachypnoea, hypotension, and altered mental status) [10] and parameters included in SIRS criteria were respiratory rate, temperature, pulse and white blood cell count [6]. These variables can be obtained readily and promptly after patients on admission.

The purpose of this study was to evaluate the predictive validity of the qSOFA and SIRS score and the established risk models containing these two parameters at different clinical settings for in-hospital mortality of SFTS patients. Meanwhile, other inflammatory parameters including white blood count (WBC) and high-sensitivity C-reactive protein (hs-CRP) were also analyzed for comparison.

## Methods

### Patients and calculation of qSOFA and SIRS score

Three hundred and twenty-one SFTS patients who were admitted to our hospital from May 2013 to July 2017 were included. Defined diagnosis of SFTS was made by detected positive SFTSV from peripheral blood samples using reverse transcription–polymerase chain reaction (RT–PCR). The following data were extracted for each patient after presentation: demographics and components of the SIRS criteria and qSOFA score (most within the first 24 h of admission). The qSOFA score includes respiratory rate ≥ 22/min, systolic blood pressure ≤ 100 mmHg, and abnormal mental status. SIRS criteria include respiratory rate > 20/min; temperature > 38 °C or < 36 °C; pulse > 90 beats/min; and white blood cell count > 12,000/μL or < 4000/μL [6]. Distribution of different qSOFA and SIRS score of all patients, survivors and nonsurvivors was measured and qSOFA score and SIRS score ≥ 2 was regarded as high. The predictive risk models for prognosis in several subgroups were constructed based on regression coefficients of the independent risk factors of outcome obtained from multi-factorial logistic regression analysis.

This study was conducted according to the Helsinki II Declaration and was approved by the ethics committee of Infectious disease hospital of Yantai. Written informed consent was obtained from the patients.

### Statistical analysis

Data are presented as numbers (percentages) and mean with standard deviation. The student *t* test or Mann–Whitney *U* test was used for the comparison of variables with normal or abnormal distribution between the survival and nonsurvival groups. Proportions of variables between groups were compared using χ ^2^ test. Independent risk factors associated with mortality were derived using multivariate logistic regression analysis. Variables that have been demonstrated as independent risk factors in our previous study [3] and those inflammatory indices such as WBC and hs-CRP, and qSOFA and SIRS score were included in a multivariate logistic regression model. Risk score models based on independent risk factors at different clinical settings were developed to reflect the underlying risk of a patient developing a fatal outcome. The discrimination of the predictive power of the independent risk factors and models were evaluated using the area under the receiver operating characteristic curve (AUC). Sensitivity (SEN), specificity (SPE), negative predictive value (NPV), positive predictive value (PPV) positive likelihood ratio (LR+) and negative likelihood ratio (LR-) were obtained using MedCalc software for each score. Cutoff values were chosen with the highest Youden index and the optimal ones the software provided. The Kaplan-Meier survival analysis was utilized to compare the cumulative risk for death in high-risk and low-risk groups according to cutoff values of the model score obtained from ROC analysis, and the significance of difference was tested with the log-rank test. Analyses were conducted using the SPSS, version 23.0, software (IBM, Armonk, NY, USA). *p* values < 0.05 were considered significant.

## Results

### Demographics and baseline qSOFA and SIRS scores in survivals and nonsurvivals

**Fig. 1:**
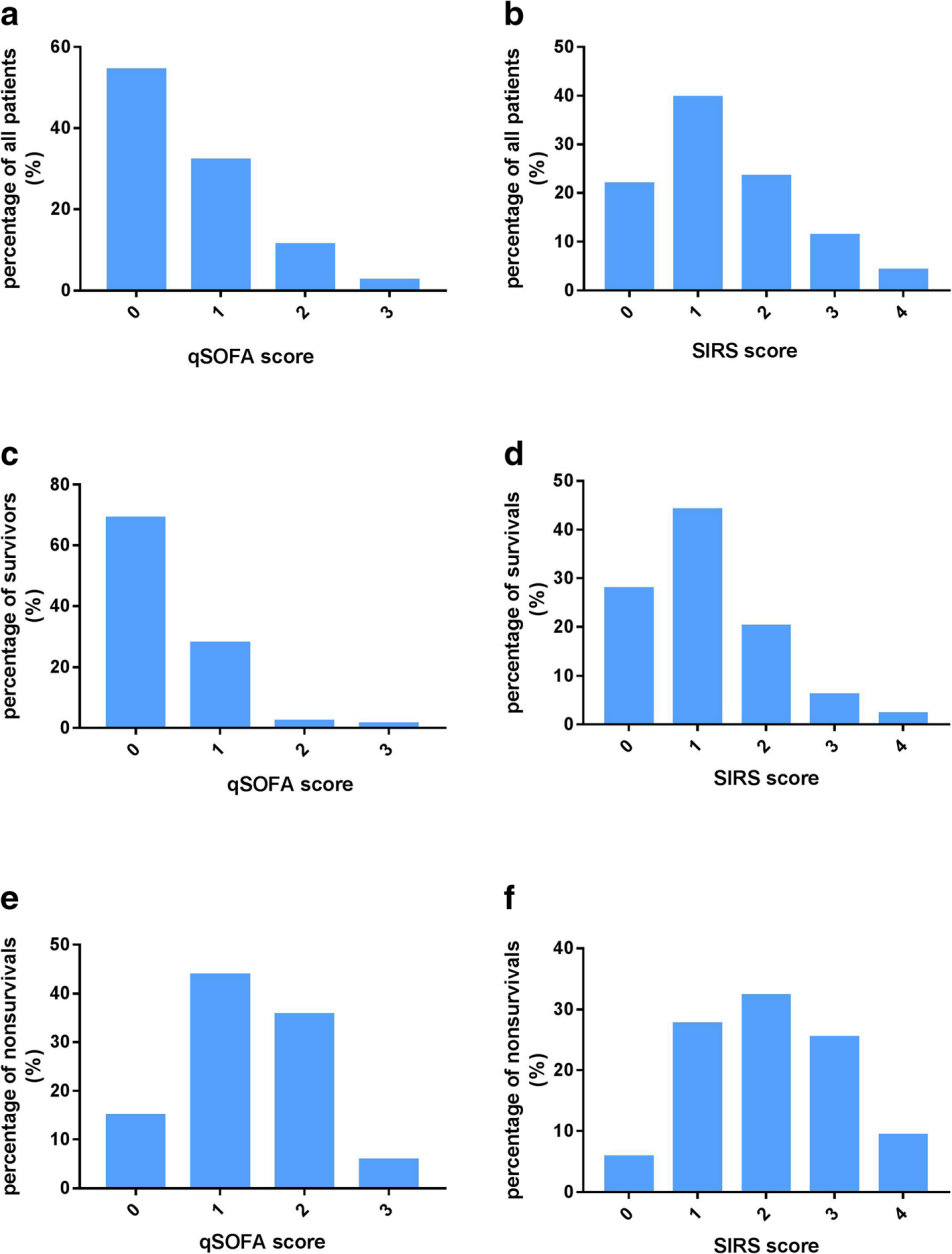
Patients number percentage with different qSOFA scores and SIRS scores. (**a**) Number distribution with qSOFA scores in all patients. (**b**) Number distribution with SIRS scores in all patients. (**c**) Number distribution of qSOFA scores in survivors. (**d**) Patients number distribution with SIRS scores in survivors. (**e**) Patients number distribution of qSOFA scores in nonsurvivors. **f** Patients number distribution of SIRS scores in nonsurvivors

Of 370 suspected SFTS patients who were admitted to our hospital from May 2013 to July 2017, 321 were confirmed by detected positive SFTSV and were included in this study. Mean age of all included patients was 63.8 ± 11.2 years. Eighty seven patients (27.1%) died during hospitalization who were older than those who survived (70.6 ± 9.2 vs 61.3 ± 10.8 years, *p* < 0.0001). Percentage of male patients in nonsurvivors was greater than in survivors (*p <* 0.05). Proportions of patients with qSOFA and SIRS score > 2 were dramatically increased in nonsurvivors compared with survivors (*p* < 0.0001). Hospital stays and biochemical parameters had significant differences between the two groups. Data are shown in Table 1 and Fig. 1.

**Table 1:** Demographics and baseline biochemical parameters, qSOFA and SIRS scores in survivors and nonsurvivors [median (IQR) or ($\overline{x}\pm \mathrm{SD}$)] of SFTS patients

Parameters	All patients	Survivors	nonsurvivors	*P* value
N, (%)	321(100)	234 (72.9)	87 (27.1)	< 0.001
Age (year), mean ± SD	63.8 ± 11.2	61.3 ± 10.8	70.6 ± 9.2	< 0.0001
Age < 60, n (%)	111(34.6)	97 (41.5)	14 (16.1)	< 0.001
Age ≥ 60, n (%)	210 (65.4)	137 (58.6)	73 (83.9)	< 0.0001
Male, n (%)	170(53)	116(49.6)	54(62.1)	< 0.05
qSOFA score ≥ 2, n (%)	44 (13.7)	8 (3.4)	36(41.4)	< 0.0001
SIRS score ≥ 2, n (%)	124(38.6)	66 (28.2)	58(66.7)	< 0.0001
PCT ≥ 0.05, n (%)	114/253(44.9)	91/185(49.2)	54/68(79.9)	< 0.0001
0.5 < PCT < 2, n (%)	50 (19.8)	8 (4.3)	42 (61.8)	< 0.0001
PCT ≥ 2, n (%)	19 (7.5)	7(3.8)	12 (17.7)	< 0.0001
Time in-hospital (days) (IQR)	11(5–15)	15(11–18)	4(3–5)	< 0.0001
plasma lactate (mmol/L) (IQR)	1.2(1–1.78)	1.1(0.9–1.4)	1.7(1.23–2.78)	< 0.0001
WBC (× 10^9^/L) (IQR)	3.15(1.9–5.0)	3.1(1.9–4.3)	3.5(1.8–6.6)	> 0.05
AST (U/L) (IQR)	183(86.6–365.5)	155(83–326.5)	428.6(170.6–1019)	< 0.0001
sCr (μmol/L) (IQR)	65.8(57.6–81.0)	65.1(54.2–74.4)	79.4(65.7–126.2)	< 0.0001
Hs-CRP (mmol/L) (IQR)	3.8(1.44–11.4)	1.8(1.26–5.7)	0.36(0.11–1.02)	< 0.0001

*qSOFA* quick sequential organ failure assessment, *SIRS* systemic inflammatory response syndrome, *PCT* Procalcitonin, *WBC* white blood cell, *AST* aspartate aminotransferase, *sCr* serum creatinine, *hs-CRP* high-sensitivity C-reactive protein

### Predictive values of independent risk factors, and established risk score models for in-hospital mortality of SFTS patients

#### Predictive values of independent risk factors and risk models for all patients

**Fig. 2:**
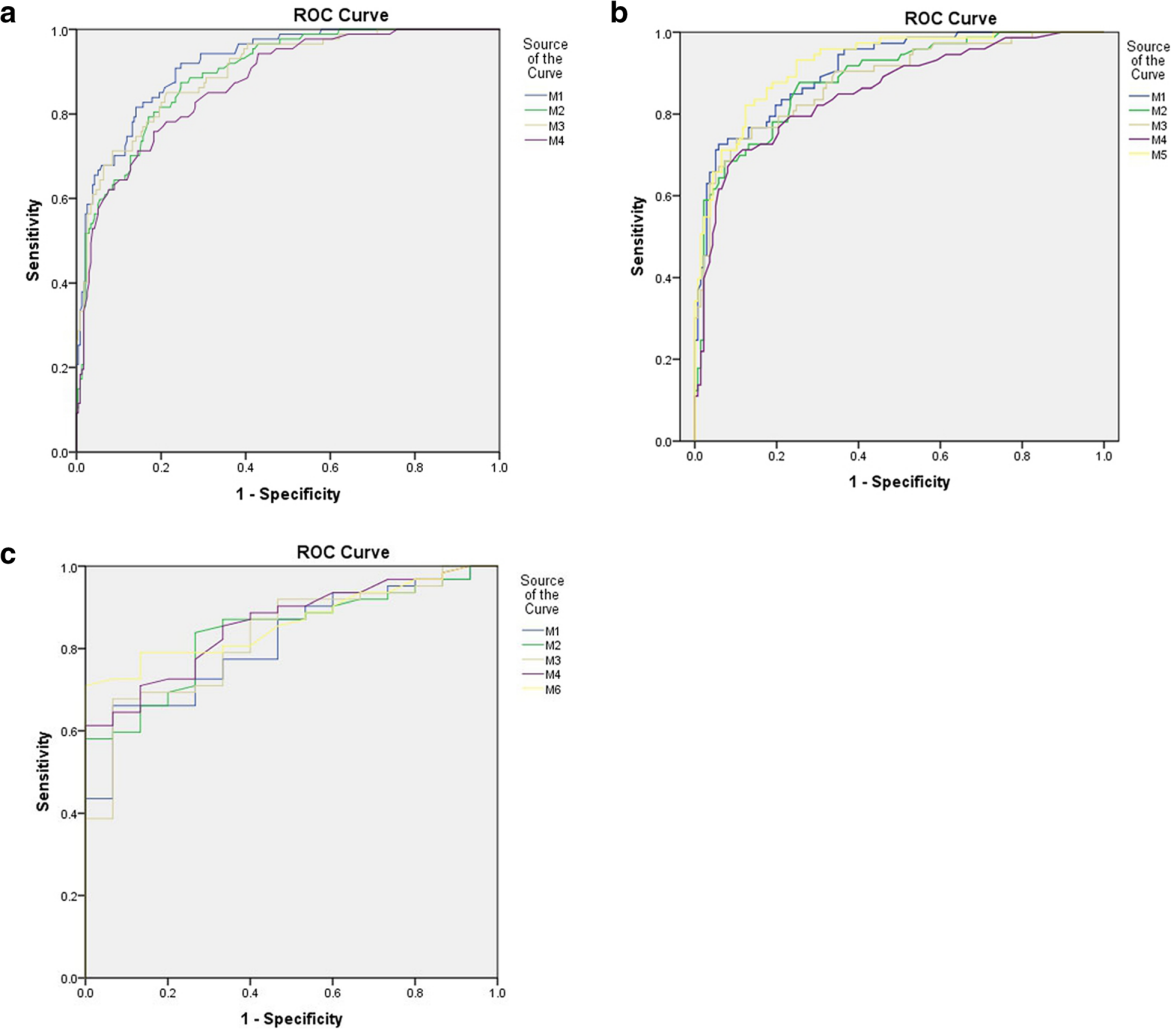
(**a**) AUCs of M1, M2, M3 and M4 for in-hospital mortality in all patients. (**b**) AUCs of M1, M2, M3, M4 and M5 for for in-hospital mortality for patients at age ≥ 60 years. (**c**) AUCs of M1, M2, M3, M4 and M6 for for in-hospital mortality for patients enrolled in ICU

**Fig. 3:**
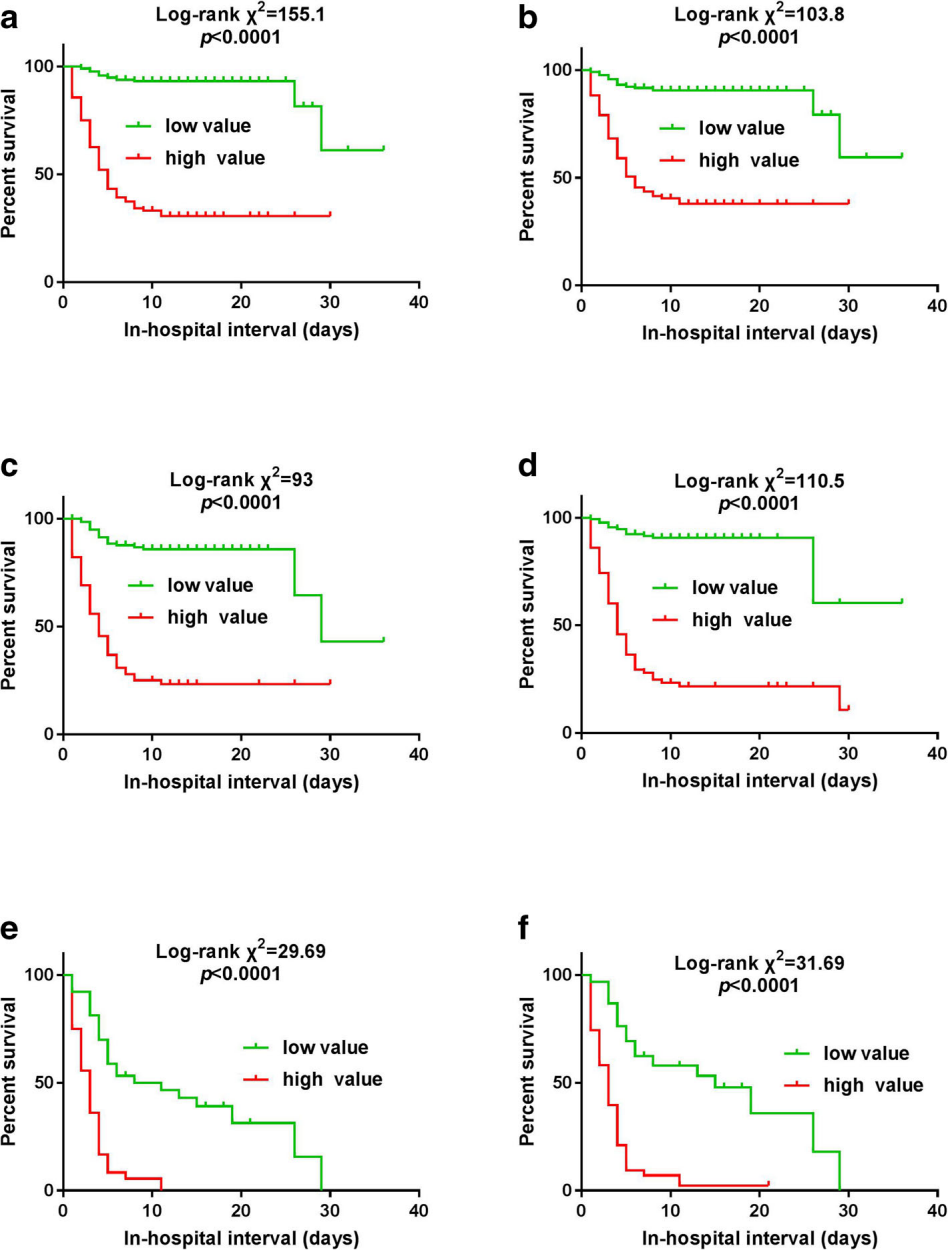
(**a**) Survival curve for all patients at cutoff value of > 9.22 of M1. (**b**) Survival curve for all patients at cutoff value of > 8.034 of M4. (**c**) Survival curve for patients of ≥60 years at cutoff value of > 8.64 of M4. (**d**) Survival curve for patients of ≥60 years at cutoff value of > 13.68 of M5. (**e**) Survival curve for patients enrolled in ICU at cutoff value of > 8.57 of M4. (**f**) Survival curve for patients enrolled in ICU at cutoff value of > 6.42 of M6

Multivariate logistic regression analysis showed that age, AST, qSOFA and SIRS scores were the independent risk factors for in-hospital mortality of all SFTS patients. AUCs (95% CI), cutoff values, SEN, SPE, PPV and NPV of these factors for the prediction of in-hospital mortality are included in Table 2. Based on values and regression coefficient of these risk factors, a risk score model was constructed as M1 = 0.102 × age+ 0.002 × AST + 1.296 × qSOFA score+ 0.486 × SIRS score. AUC (95% CI) of M1 was 0.919 (0.883–0.946) with odd ratio (OR) (95% CI) = 2.95 (2.308–3.771) at the cutoff value of 9.22 (Table 2, Fig. 2a). Kaplan-Meier survival analysis showed a strong difference between high-risk and low-risk groups (log-rank test, χ^2^ = 1551.1, *p* < 0.0001) (Fig. 3a).

**Table 2:** Predictive values of independent risk factors and established models containing different risk factors for in-hospital mortality of all SFTS patients

Parameters	OR (95%)	*P* value	Cutoff value	AUC (95%	SEN	SPE	PPV	NPV	LR+	LR-
Age (years)	1.107 (1.039–1.176)	< 0.0001	> 66	0.742 (0.69–0.789)	70.1	68.4	45.2	86	2.22	0.44
			> 74		39.1	89.3	57.6	79.8	3.66	0.68
AST (U/L)	1.002 (1.000–1.003)	0.01	> 205	0.794 (0.729–0.849)	77.4	67	45.8	89.1	2.34	0.34
			> 735		38.1	96.1	78	81.2	9.86	0.64
qSOFA	3.654 (1.488–6.378)	< 0.0001	> 0	0.818 (0.774–0.858)	85.1	68.8	50.3	95.2	2.73	0.22
			> 1		41.4	95.6	81.8	81.6	12.1	0.61
SIRS	1.625 (1.025–2.581)	0.039	> 1	0.740 (0.688–0.787)	66.7	71.8	46.8	81.3	2.36	0.46
			> 2		34.5	91.9	61.2	79	4.25	0.71
M1	2.95 (2.308–3.771)	< 0.0001	> 9.22	0.919 (0.883–0.946)	81.6	85.9	68.3	92.6	5.79	0.21
			> 10.27		65.2	95.7	85.1	88.2	15.3	0.36
M2	3.122 (2.401–4.033)	< 0.0001	> 8.034	0.894 (0.855–0.925)	87.4	75.2	56.7	94.1	3.52	0.17
			> 9.942		51.7	91.9	90	84.5	24.2	0.349
M3	2.986 (2.324–3.837)	< 0.0001	> 8.25	0.899 (0.861–0.930)	85.1	78.6	59.7	93.4	3.98	0.19
			> 9.06		71.3	91.5	75.6	89.5	8.34	0.31
M4	3.26 (2.437–4.297)	< 0.0001	> 8.034	0.872 (0.830–0.906)	85.9	81.6	60.6	90.1	4.13	0.3
			> 8.44		63.2	91.0	72.4	86.9	7.04	0.4

*OR* odd ratio, *CI* confidence interval, *AUC* area under ROC curve, *AST* aspartate aminotransferase, *qSOFA* quick sequential organ failure assessment, *SIRS* systemic inflammatory response syndrome, *SEN* sensitivity, *SPE* specificity, *PPV* positive predictive value, *NPV* negative predictive value, *LR+* positive likelihood ratio, *LR-* negative likelihood ratio

Considering relative small regression coefficient of AST and SIRS scores, we modified the models into simpler ones as:

$$ \mathrm{M}2\ \left(\mathrm{model}\ 2\right)=0.102\times \mathrm{age}+1.296\times \mathrm{qSOFA}+0.486\times \mathrm{SIRS} $$

$$ \mathrm{M}3\ \left(\mathrm{model}\ 3\right)=0.102\times \mathrm{age}+0.002\times \mathrm{AST}+1.296\times \mathrm{qSOFA} $$

$$ \mathrm{M}4\ \left(\mathrm{model}\ 4\right)=0.102\times \mathrm{age}+1.296\times \mathrm{qSOFA} $$

These three models had comparable predictive power and had a relative less power than M1 (Table 2, Fig. 2).

Survival analysis demonstrated a strong statistical significance between high and low-risk groups of M1 and M4 (selection based on the highest AUC and OR values) (*p* < 0.0001) (Fig. 3a, b).

#### Predictive values of independent risk factors and risk models for patients with age ≥ 60 years

The four models also had high predictive values for in-hospital mortality in those patients at age ≥ 60 years (Table 3). Logistic regression analysis indicated that age, plasma lactate, serum AST and hs-CRP levels, and qSOFA and SIRS scores were the independent risk factors for in-hospital mortality in this group of patients. Based on these factors, we established another risk score model:

$$ \mathrm{M}5=0.127\times \mathrm{age}+0.953\times \mathrm{lactate}+0.002\times \mathrm{AST}+0.056\times \mathrm{Hs}-\mathrm{CRP}+0.86\times \mathrm{qSOFA}+0.866\times \mathrm{SIRS} $$

**Table 3:** Predictive values of independent risk factors and established models containing different risk factors for in-hospital mortality of patients with age ≥ 60 years

Parameters	OR (95% CI)	*P* value	Cutoff value	AUC (95% CI)	SEN	SPE	PPV	NPV	LR+	LR-
Age (years)	1.135 (1.037–1.243)	0.006	> 72	0.706 (0.640–0.767)	56.2	74.5	53.9	76.1	2.2	0.59
			> 74		46.6	81.8	57.6	74.2	2.55	0.65
lactate (mmol/L)	2.592 (1.06–6.34)	0.037	> 1.17	0.757 (0.674–0.828)	85.5	52	56.6	83	1.78	0.28
			> 2.1		38.2	94.7	84	67.6	7.16	0.65
Hs-CRP (mmol/L)	1.058 (1.007–1.11)	0.024	> 6.3	0.729 (0.69–0.789)	81.7	54.7	48.3	85.2	1.81	0.33
			> 3.14		25.4	95.6	75	71.2	5.79	0.78
AST (U/L)	1.002 (1.000–1.003)	0.01	> 203	0.779 (0.717–0.834)	78.8	66.4	54.9	85.8	2.35	0.32
			> 722		39.4	95.6	82.4	75.3	9	0.63
qSOFA	2.364 (1.142–4.89)	0.02	> 0	0.818 (0.759–0.867)	83.6	71.5	61	89.1	2.94	0.23
			> 1		39.7	97.1	87.9	75.1	13.6	0.62
SIRS	2.377 (1.025–2.581)	0.008	> 1	0.778 (0.716–0.833)	68.5	76.6	61	82	2.93	0.41
			> 2		35.6	94.9	78	73.4	6.97	0.68
M1	3.144 (2.319–4.263)	< 0.0001	> 10.272	0.909 (0.862–0.944)	72.6	94.2	86.9	86.6	12.43	0.29
M2	3.508 (2.506–4.910)	< 0.0001	> 8.544	0.886 (0.835–0.926)	87.7	75.5	64.6	91.9	3.43	0.17
			> 9.942		58.9	97.8	26.9	0.42	26.9	0.42
M3	3.11 (2.286–4.231)	< 0.0001	> 9.3	0.883 (0.831–0.923)	73.97	89.78	79.4	86.6	7.24	0.29
M4	3.533 (2.494–5.005)	< 0.0001	> 8.64	0.885 (0.801–0.900)	69.86	89.78	78.5	84.8	6.84	0.34
M5	2.508 (1.951–3.223)	< 0.0001	> 13.68	0.924 (0.879–0.956)	82.2	87.6	77.9	90.2	6.62	0.2

*OR* odd ratio, *CI* confidence interval, *AUC* area under ROC curve, *AST* aspartate aminotransferase, *hs-CRP* high-sensitivity C-reactive protein, *qSOFA* quick sequential organ failure assessment, *SIRS* systemic inflammatory response syndrome, *SEN* sensitivity, *SPE* specificity, *PPV* positive predictive value, *NPV* negative predictive value, *LR+* positive likelihood ratio, *LR-* negative likelihood ratio

This model (M5) has the highest predictive value among these models for in-hospital mortality in this group of patients (Table 3, Fig. 2b).

Survival analysis manifested a profound statistical significance between high and low-risk groups of M4 and M5 (selection based on the highest AUC and OR values) *p* < 0.0001) (Fig. 3c, d).

#### Predictive values of independent risk factors and risk models for patients in ICU

The former four models also have high predictive values for in-hospital mortality in those patients enrolled in ICU at admission or transferred to (Table 4, Fig. 2c).

**Table 4:** Predictive values of independent risk factors and established models containing different risk factors for in-hospital mortality of patients enrolled in intensive care unit (ICU)

Parameters	OR (95% CI)	*P* value	Cutoff value	AUC (95% CI)	SEN	SPE	PPV	NPV	LR+	LR-
age (years)	1.073 (1.008–1.142)	0.028	> 68	0.685 (0.570–0.787)	62.9	73.3	90.7	32.4	2.36	0.51
qSOFA	6.299 (1.932–20.53)	0.02	> 0	0.828 (0.726–0.905)	79.03	73.3	92.5	45.8	2.96	0.29
M1	1.875 (1.287–2.732)	0.001	> 10.15	0.815 (0.710–0.894)	61.1	93.3	97.6	40	9.92	0.36
			> 7.84		93.6	40	86.6	60	1.56	0.16
M2	2.419 (1.444–4.502)	0.001	> 9.54	0.838 (0.737–0.912)	58.1	100	100	36.6		
			> 7.986		87.1	66.7	91.5	55.6	2.61	0.19
M3	2.039 (1.342–3.099)	0.001	> 8.83	0.827 (0.724–0.904)	67.7	93.3	97.7	41.2	10.16	0.35
M4	2.927 (1.611–5.318)	< 0.0001	> 8.57	0.859 (0.761–0.928)	61.3	100	100	38.5		0.39
M6	2.72 (1.573–4.705)	< 0.0001	> 6.42	0.865 (0.767–0.932)	70.8	100	100	45.5		0.29

*OR* odd ratio, *CI* confidence interval, *AUC* area under ROC curve, *SEN* sensitivity, *SPE* specificity, *PPV* positive predictive value, *NPV* negative predictive value, *LR+* positive likelihood ratio, *LR-* negative likelihood ratio

Univariate logistic regression showed that age and qSOFA score were the independent risk factors for mortality of this subgroup of patients. Multivariate logistic regression analysis demonstrated that qSOFA score was the only independent risk factor for mortality of these patients. Based on these two parameters we built another model that has the same index of M4 with different regression coefficient:

$$ \mathrm{M}6\ \left(\mathrm{model}\ 6\right)=0.071\times \mathrm{age}+1.877\times \mathrm{qSOFA} $$

This model has the highest predictive value among these models for in-hospital mortality in this subgroup of patients (Table 4, Fig. 2c).

Survival analysis showed a significant statistical difference between high and low-risk groups of M4 and M6 (selection based on the highest AUC and OR values) *p* < 0.0001) (Fig. 3e, f).

Of note, no multi-colinearity between SOFA and SIRS were detected in three models cotaining these two parameters.

## Discussion

SFTS is a severe emerging virus infectious disease which is a great life-threat to people locally and globally. In this study, we evaluated the varied predictive validity of qSOFA score, SIRS score and established risk models in different cohort and clinical settings of SFTS patients. Our results showed that qSOFA score was the only common index associated with in-hospital mortality of SFTS patients in different settings, and was the only independent risk factor for patients admitted in ICU.

M1 composed of age, AST, qSOFA and SIRS score has the highest predictive value for in-hospital mortality in all patients (Table 2, Fig. 2a) and M5 has the highest predictive power for those of age ≥ 60 years (Table 3, Fig. 2b, c) and relative low predictive power in those enrolled in ICU (Table 2). However the modified simpler ones without AST and SIRS scores have higher predictive validity than M1 in these patients. This suggests that different models have diverse predictive values in different clinical settings. And clinical manifestation indices without biochemical parameters can also have high predictive efficacy for prognosis of diverse cohort SFTS patients.

Although model 5 (M5) holds the highest predictive validity for patients at age ≥ 60 years, the complexity of M5 composition limited its application to initial admission when some parameters are not at hand. Survival analysis illustrated profound differences between low risk and high risk groups of M4 and M5 (*p* < 0.0001). Because of its simpler component parts, M4 can be used as a suitable risk model for predicting prognosis of SFTS patients at age ≥ 60 years.

SOFA score has been demonstrated the optimal indicator for sepsis in patients enrolled in ICU [5]. Its utility is restricted outside the ICU because many SOFA variables, such as cardiorespiratory, neurologic organ dysfunction are not measured routinely. qSOFA requires only a clinical examination and physiological parameters, including respiratory rate, mental status, and systolic blood pressure, and therefore is readily applicable which is especially valuable in resource-limited settings [7].

Though SIRS score is inferior to qSOFA score for the prediction of adverse outcome in our results, it adds the advantage of predictive value together with age and /or other parameters compared with the simpler qSOFA score. A recent study suggested that at least 2 of qSOFA score could be an alternative parameter for septic patients fulfilled the SIRS criteria [11]. In our results we show that qSOFA score of more than zero can predict adverse outcome of SFTS with high sensitivity which indicates that qSOFA score as a synthetical index can be used combining with age or used solely as a sensitive indicator for predicting prognosis of SFTS.

As a systemic virus infectious disease with overall dysfunction of cellular and humoral immunity [12, 13], SFTS patients were susceptible to bacterial and fungal infection. Procalcitonin (PCT) is a useful parameter to guide antibiotic therapy in severe sepsis patients [14]. While it is not an independent risk factor associated with in-hospital mortality in our results. The reason may be that on one hand it presents as a semiquantitative result, and on the other hand, PCT is not specificity to secondary bacterial infections of virus infection [15]. Other inflammatory parameters such as Hs-CRP and WBC are not independent risk factors for in-hospital mortality except for aged patients which suggest that a single index can not reflect disease status and predict disease outcome.

There are several limitations in this study. First, it is a single center study with a relatively small number of patients which may affect the predictive efficacy of the indices and models. Second, SFTSV infection can influence the number of WBC, therefore impact the accuracy of SIRS score which may influence the predictive power of established models containing SIRS score. Third, the models need further validation in prospective studies including large number of patients.

## Conclusions

In all, risk score models containing qSOFA have high predictive value for in-hospital mortality of SFTS patients with different clinical settings.

## Data Availability

The datasets used and/or analysed during the current study are available from the corresponding author on reasonable request.

## Abbreviations

ALT: Alanine aminotransferase
AST: Aspartate aminotransferase
AUCs: Area under ROC curve
BUN: Blood urea nitrogen
hs-CRP: High-sensitivity C-reactive protein
ICU: Intensive care unit
LR+: Positive likelihood ratio
LR-: Negative likelihood ratio
NPV: Negative predictive value
PPV: Positive predictive value
PCT: Procalcitonin
qSOFA: Quick sequential organ failure assessment
ROC: Receiver-operating characteristic
RT–PCR: Reverse transcription–polymerase chain reaction
SEN: Sensitivity
SPE: Specificity
SIRS: Systemic inflammatory response syndrome
sCr: Serum creatinine
SFTS: Severe fever with thrombocytopenia syndrome
SFTSV: SFTS virus
WBC: White blood cell
